# HIV-1 Nef Signaling in Intestinal Mucosa Epithelium Suggests the Existence of an Active Inter-kingdom Crosstalk Mediated by Exosomes

**DOI:** 10.3389/fmicb.2017.01022

**Published:** 2017-06-08

**Authors:** Cristina Felli, Olimpia Vincentini, Marco Silano, Andrea Masotti

**Affiliations:** ^1^Gene Expression – Microarrays Laboratory, Bambino Gesù Children’s Hospital – Istituto di Ricovero e Cura a Carattere ScientificoRome, Italy; ^2^Unit of Human Nutrition and Health, Department of Veterinary Public Health and Food Safety, Istituto Superiore di Sanità – Italian National Institute of HealthRome, Italy

**Keywords:** HIV-1, Nef, Caco-2, intestinal mucosa, tight junctions, exosomes, microRNAs

## Abstract

The human intestinal mucosal surface represents the first defense against pathogens and regulates the immune response through the combination of epithelial cell (EC) functions and immunological factors. ECs act as sensors of luminal stimuli and interact with the immune cells through signal–transduction pathways, thus representing the first barrier that HIV-1 virus encounters during infection. In particular, the HIV-1 Nef protein plays a crucial role in viral invasion and replication. Nef is expressed early during viral infection and interacts with numerous cellular proteins as a scaffold/adaptor. Nef is localized primarily to cellular membranes and affects several signaling cascades in infected cells modulating the expression of cell surface receptors critical for HIV-1 infection and transmission, also accompanied by the production of specific cytokines and progressive depletion of CD4^+^ T cells. At the intestinal level, Nef contributes to affect the mucosal barrier by increasing epithelial permeability, that results in the translocation of microbial antigens and consequently in immune system activation. However, the pathological role of Nef in mucosal dysfunction has not been fully elucidated. Interestingly, Nef is secreted also within exosomes and contributes to regulate the intercellular communication exploiting the vesicular trafficking machinery of the host. This can be considered as a potential inter-kingdom communication pathway between virus and humans, where viral Nef contributes to modulate and post-transcriptionally regulate the host gene expression and immune response. In this mini-review we discuss the effects of HIV-1 Nef protein on intestinal epithelium and propose the existence of an inter-kingdom communication process mediated by exosomes.

## Introduction

### The Gastrointestinal Mucosal Barrier and Cell–Cell Communication Mechanisms

Gastrointestinal (GI) tract represents the largest mucosal area in the human body. Mucosal surfaces biophysically separate the human body from the outer environment through layers of epithelial cells (ECs) closely joined by intercellular tight junctions (TJs). In the gut, ECs in addition to soluble mediators, can release a wide variety of proteins, lipids, mRNAs and microRNAs contained within secreted membrane vesicles that are formed in endosomal compartments. The exchange between cells is essential to ensure an efficient immune response. Exosomes, characterized by specific endosomal and plasma membrane proteins (i.e., acetylcholinesterase, Alix, and CD45) fuse with membranes of neighboring cells releasing their content and triggering a variety of responses in target cells (Simons and Raposo, 2009; Ohno et al., 2013). Apical secretion of exosomes into the lumen may modulate the function of distant cells along the gastrointestinal tract, through the delivery of antimicrobial product. The basolateral release of exosomes into the mucosa may also regulate local innate responses. Moreover, EC-derived exosomes released into the mucosa may be taken up by mucosal DCs and transported to the lymph nodes, where their contents can influence adaptive immune responses. This response may direct CD4^+^ T cell populations either to be tolerogenic or effector cells, respectively, in homeostasis and during intestinal mucosa infection and microbial invasion (Smythies and Smythies, 2014). Therefore, exosomes may play a crucial role in cell–cell communication (Gould et al., 2003).

### HIV-1 Infection

**FIGURE 1:**
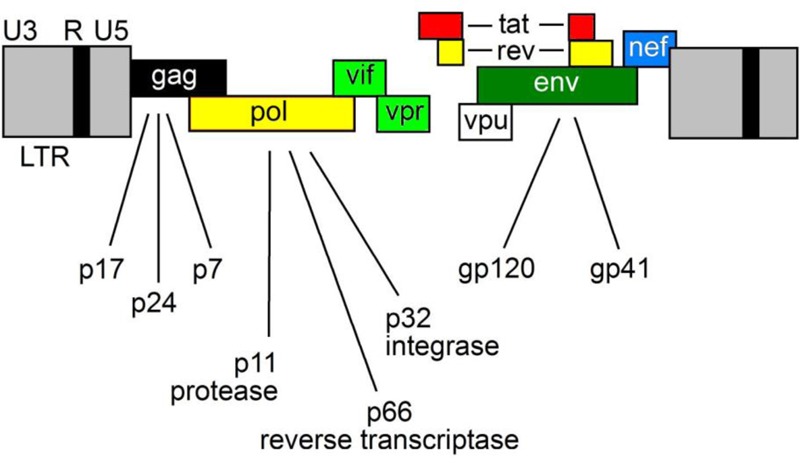
The HIV-1 genome. HIV-1 encodes three major genes, 5′-gag-pol-env-3′ encoding structural, accessory and regulatory proteins.

Epithelial cells provide a defense against infection and prevent the access of commensal bacteria, pathogens and toxins into the sub-epithelial tissues. Despite this, most HIV-1 infections occur by mucosal transmission. The lymphoid tissue of the gastrointestinal tract forms the gut-associated lymphoid tissue (GALT), which is the preferred target for HIV-1. In fact, GALT provides an advantageous microenvironment for viral replication owing to the presence of CCR5-expressing CD4^+^ T cells. Once HIV-1 has crossed the ECs and infected the host, it replicates in the mucosal sites. Then, its dissemination leads to an extensive CD4^+^ T cells destruction. This process leads to an enteropathy characterized by partial villous atrophy and an increased number of apoptotic ECs (Epple and Zeitz, 2012). The derangement of the GI tract leads to the damage of the mucosal barrier that, in turn, allows the entry of microbial products (i.e., bacterial lipopolysaccharides) into the host circulation and the activation of local and systemic immune response. ECs trigger inflammatory responses through pattern recognition receptors and induce the synthesis of cytokines, chemokines and antimicrobial peptides that activate innate immune responses. This process is advantageous for HIV-1, as an inflammatory cascade at the mucosal level is required for the establishment of productive viral infection. The mechanisms that allow the binding and entry of HIV-1 into the host cell determine the ability of virus to evade the host immune response. First, the glycoprotein gp120, located on the surface of virions, binds to its primary cellular receptor CD4, expressed on several immune and non-immune cells including T-helper lymphocytes, macrophages, dendritic cells (DCs) and brain-resident microglia. This receptor recognition event triggers a conformational change that enables gp120 to bind the chemokine receptor CCR5 or CXCR4. Then, a further conformational change induces the membrane glycoprotein gp41 to mediate the fusion of viral and cellular membranes. Finally, once the viral core is released into the cytoplasm of the host cell, a reverse transcription reaction takes place in order to produce a double-stranded DNA that will be transported and integrated into the nucleus. With this tricky system, the provirus replicates whenever the host cell replicates. The HIV-1 genome encodes Gag (capsid), Pol (polymerase) and Env (envelop) polyproteins that intervene to assemble viral RNAs and to release and mature new infectious virions (**Figure 1**).

Moreover, HIV-1 encodes accessory proteins such as Nef, Vpr, Vif and Vpu and the two regulatory proteins Tat and Rev (Wilen et al., 2012a,b). Nef gene is located at the 3′-end of the viral genome and partially overlaps with the long terminal repeat (LTR).

Therefore, HIV-1 uses a complex machinery to deliver its genome into the host cells and also affects the susceptibility to infection.

### HIV-1 Nef Protein Localization

To understand in more details the pathological changes occurring in the GI tract after HIV-1 infection, many studies employing *in vitro* models have been carried out. The treatment of cultured intestinal epithelial T84 colon cells with supernatants collected from HIV-infected macrophages, induced epithelial apoptosis, decreased *trans*-epithelial electrical resistance (TEER) and disruption of TJ proteins such as claudin-1,2,4 and zonula occludens-1 (ZO-1), demonstrating that HIV-1 is able to produce factors able to perturb the TJ of intestinal epithelium (Nazli et al., 2010).

During infection, damages of epithelial barrier may be induced by viral factors. Some of these effects can be mediated by gp120 or Tat, which accelerate the apoptosis of human intestinal lamina propria T cells or enterocytes, respectively (Boirivant et al., 1998; Buccigrossi et al., 2011). Nef, involved in AIDS pathogenesis and disease progression, may affect the integrity of intestinal epithelium. In fact, it is expressed in the early stage after virus infection, anchored with intracellular membranes through its N-terminal end myristoylated (Bentham et al., 2006). Nef is also present in circulation such as in the serum and plasma of HIV-1 infected patients (Fujii et al., 1996). However, although primarily localized to cellular membranes, (Olivetta et al., 2016) Nef is secreted by infected cells within membrane vesicles (although no secretion pathways have been identified), thereby extending its immune regulation beyond infected cells. In fact, Nef is found associated with markers that normally characterize exosomes (i.e., Alix, CD45, and AChE) showing that they incorporate the protein (Ali et al., 2010; Lenassi et al., 2010; Konadu et al., 2015). Many studies demonstrated that HIV-1 infected CD4^+^ T lymphocytes release exosomes able to activate bystander quiescent human CD4^+^ T lymphocytes, thus stimulating viral spread. HIV-1 replication was observed in quiescent CD4^+^ T lymphocytes also when they were treated with exosomes derived from cells expressing Nef, suggesting its involvement in the mechanism (Arenaccio et al., 2014). Thus, Nef may regulate intercellular communication through exosomes.

### HIV-1 Nef Protein Function

Nef has been reported to alter the immune response through many different biological processes. Depending on its intracellular localization, Nef may exert multiple effects such as interfering with cellular signal transduction pathways or modulating the cell surface expression of many membrane-associated proteins in infected cells (Greenway et al., 2003; Quaranta et al., 2009). In particular, Nef can affect the T-cell signaling pathways and increase the infectivity of new virus particles released from the cell (Fujii et al., 1996; Aiken, 2015). Nef down-regulates MHC class I, reducing the recognition of HIV-infected cells by CTLs and allowing the evasion of immune surveillance (Collins et al., 1998). However, MHC class I down-regulation exposes the virus-infected cells to the attack by NK cells. Human lymphoid cells are protected from NK cell cytotoxicity primarily by HLA-C and HLA-E. Thus, HIV-1 is able to down-regulate HLA-A and HLA-B alleles from the cell surface, but not HLA-C or HLA-E. This selective down-regulation allows HIV-infected cells to avoid NK cell-mediated lysis and may represent for the virus a balance between escape from CTLs and maintenance of protection from NK cells (Aiken et al., 1994; Cohen et al., 1999). At the same time, Nef inhibits MHC class II preventing the induction of antiviral immune responses. The protein down-regulates the expression of CD4, and CD28 from T-cell surfaces, by activating endocytosis and lysosomal degradation. The effect of Nef on surface receptors may interfere with TCR signaling, reducing T cell activation to the advantage of the virus (Stumptner-Cuvelette et al., 2001; Swigut et al., 2001). Moreover, Nef inhibits lymphocyte migration by down-regulating CXCR4 and interleukin (IL) 2 receptor, by impairing the ability of the infected T-cell to respond to IL-2 and upregulating the Fas ligand expression to induce CTLs apoptosis (Greenway et al., 2003).

Nef is able to trigger the transition of DCs from immature antigen-capturing cells to more competent antigen-presenting cells, upregulating the expression of DCs surface molecules, critical for their function in the activation of both the innate and adaptive immune response. These molecules include CD1a and HLA-DR involved in the presentation of lipid and antigenic peptides to CD4^+^ T cells, respectively; CD40, which transduces activation signals; CD83, a maturation antigen for DCs; and CXCR4 (Quaranta et al., 2002). HIV-1 virus is unable to directly infect B cells, thus assuming the presence of an indirect process. Nef can be internalized from extracellular environment and accumulated in B cells in the germinal centers of infected lymphoid follicles. Nef can render B cells less responsive to CD4^+^T activation, inhibiting the switching to IgG, IgA, and IgE (Quaranta et al., 2009) and blocking the CD40 ligand. Moreover, Nef increases infectivity of produced HIV-1 virions, by preventing the incorporation of two antiviral cellular proteins, SERINC3 and SERINC5 (Rosa et al., 2015; Usami et al., 2015).

### HIV-1 Nef Protein and the Gut

The virus-host interactions within the gut likely have a significant impact on the course of a disease. Gut acts as a reservoir and the HIV-1 Nef protein has a key role in viral persistence through its multiple functions. Gene expression of viral Nef gene is also highly diversified in different areas of the intestine (van Marle et al., 2007). In a recent study, it has been determined whether Nef sequence conservation and function are separated into distinct sections within the gut and how they are related to local CTL immune responses (Lewis et al., 2013). Primary Nef variants from paired plasma and sigmoid colon biopsies from chronically infected subjects, not on therapy, were sequenced and cloned into proviral vector in which the portions of *vpu* and *env* have been deleted. Two key functions of Nef such as CD4 and MHC class I down-regulation were measured by flow cytometry. Although the measurement of interferon- gamma (IFN-γ) secretion is not a comprehensive measure of CTL function, it is, however, a good method to measure HIV-specific CTL responses. MHC class I expression was relatively well conserved by all variants regardless of location, showing a significantly reduced function. However, there was a significant down-regulation of CD4 by different variants in 5 of 7 subjects. In some gut isolates, CD4 down-regulation was not observed, whereas it was preserved in all blood isolates examined. MHC class I expression was relatively conserved in both compartments. In that study, the level of interferon-gamma (IFN-γ) released from peripheral blood mononuclear cells and colonic mucosal mononuclear cells was used as a good method to estimate HIV-specific CTL response. The reported results demonstrated that CTL responses are not compartment-dependent. Therefore, Nef may contribute to the selection of functionally distinct viral populations in gut microenvironment.

### Effects of Exogenous HIV-1 Nef Protein on Different Models of Epithelial Cells

Epithelial cells are the first layer of cells that HIV-1 encounters during the infection, independently by the way of transmission (breastfeeding or sexual transmission). Recently, the exposure effects of extracellular HIV-1 Nef on human T24, HEp-2 and Caco-2 cell lines derived from bladder, laryngeal and colonic carcinoma, respectively, has been investigated (Quaranta et al., 2011a). The expression of CCR5, CXCR4 and galactosylceramide (GalCer), three receptors on ECs surface, was evaluated by flow cytometry. The treatment with Nef resulted to increase significantly the percentage of CXCR4-expressing Caco-2 but not CXCR4-expressing HEp-2 and T24; in addition, Nef upregulated the percentage of Gal-Cer-expressing T24, but not Gal-Cer-expressing Hep-2 and T24. Nef did not alter the expression of CCR5 in any of three analyzed cell lines. However, an increased susceptibility to HIV-1 entry into Caco-2 and T24 cells has been observed. IFN-γ is the cytokine preferentially secreted by activated T lymphocytes and NK cells and is the major player of inflammation and immune responses. IFN-γ may mimic *in vitro* the mucosal inflammation, which characterizes acute HIV-1 infection. Advanced stages of HIV-1 infection are characterized by chronic inflammation, therefore the role of Nef has been also studied on cells stimulated by IFN-γ. IFN-γ significantly increased the percentage only of CCR5- and GalCer-expressing HEp-2 cells, but not that of CXCR4-expressing HEp-2 and T24 cells. Noteworthy, Nef was able to abrogate the effect of IFN-γ. Very similar results were obtained for the secretion of tumor necrosis factor alpha (TNF-α) and IL-6 by HEp-2, T24 and Caco-2 cells following exposure to Nef, IFN-γ or their combination. The obtained results demonstrate that exogenous Nef modulates the co-expression of receptors and cytokines production at the epithelial level, and that this process depends on the anatomical derivation of the cells and the inflammatory status.

### Effects of Exogenous HIV-1 Nef Protein on the Integrity of Human Intestinal Epithelium

Although numerous reports describe how HIV-1 affects mucosal immunity, the mechanisms underlying HIV-1-induced impairments of mucosal epithelial barrier are still unclear. Besides the effects observed on different cell types that are involved in immune response, a quite recent study revealed that Nef may also play a crucial role in AIDS-related gastrointestinal dysfunction, targeting ECs (Quaranta et al., 2011a). Chronic HIV-1 infection is characterized by increased intestinal permeability and enteropathy, and a chronic activation of the immune system, which significantly predicts the disease progression. The most suitable *in vitro* model for this kind of studies is represented by human intestinal Caco-2 cell line, able to spontaneously differentiate in long-term culture, resulting in the formation of a cell monolayer that encloses several morphological and functional characteristics of mature enterocytes (Sambuy et al., 2005). Confocal laser scanning microscopy allowed to demonstrate that exogenous Nef was efficiently internalized into Caco-2 cells independently from IFN-γ treatment. Interestingly, in IFN-γ-treated Caco-2 cells, Nef was internalized and retained for 24 h suggesting that IFN-γ may have a positive effect in extending Nef stability into cells. The TEER of differentiated Caco-2 cell line, grown on polycarbonate inserts, was also analyzed to measure the monolayer integrity. IFN-γ reduced TEER (i.e., increased permeability) and FITC-dextran assay confirmed this finding. Authors concluded that exogenous Nef, similarly to IFN-γ, significantly up-regulated epithelial permeability of Caco-2 differentiated monolayer, but did not significantly modulate TEER. On the contrary, when used in combination with IFN-γ, Nef was ineffective and did not alter TEER levels. Since TJ regulate paracellular permeability, authors examined the effect of Nef, IFN-γ or their combination on the expression of two TJ proteins: ZO-1 and occludin. Singularly, Nef and IFN-γ induced a reduction in the expression levels of both TJ proteins, while the effect of their combination was not completely understood.

**FIGURE 2:**
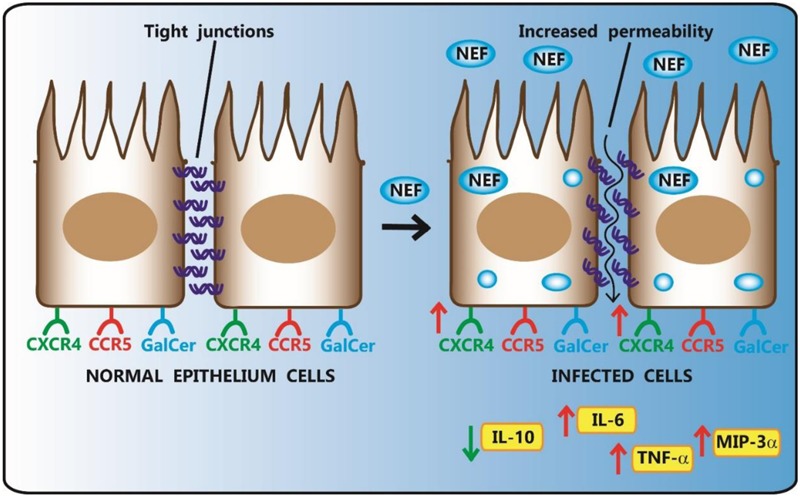
Effect of HIV-1 Nef on human intestinal epithelial Caco-2 cell lines. HIV-1 Nef, taken up by Caco-2 cells, up-regulates epithelial permeability of monolayer and the percentage of CXCR4-expressing Caco-2 cells. Following Nef exposure, ECs increase the release of TNF-α, IL-6 and MIP-3α, while decrease IL-10 secretion.

Intestinal ECs respond to tissue damage secreting cytokines and chemokines. Following the treatment with IFN-γ, ECs increased the expression of TNF-α and decreased that of the anti-inflammatory IL-10, while maintained unaltered the secretion of IL-6, macrophage inflammatory protein-3-alpha (MIP-3α) and IL-8. Moreover, Nef exposure determined the release of TNF-α, IL-6 and MIP-3α, whereas IL-10 was down-regulated. Interestingly, the treatment with Nef and IFN-γ inhibited completely the IFN-γ-induced upregulation of TNF-α and the down-regulation of IL-10 (**Figure 2**).

Finally, Nef has been reported to have many other effects also in other related pathways. For example, the differentiation of Caco-2 cell line is characterized by an increase of polyunsaturated fatty acids, critical in immunity and inflammation. Nef can inhibit the IFN-γ-induced arachidonic acid cascade or the impairment of intestinal ECs, possibly prolonging cell survival. Finally, the co-stimulation with Nef and IFN-γ did not affect the percentage of apoptotic cells, that otherwise are increased with IFN-γ alone.

### Inter-Cellular Transmission of HIV-1 Nef Protein

Many functions of HIV-1 Nef protein have been attributed mainly considering its association with intracellular membranes or virions. However, HIV-1 infection induces the formation of exosomes and their secretion from infected host cells. Thus, Nef may be secreted from infected cells in association with multivesicular bodies (MVBs) that fuse with plasma membrane resulting in the generation of exosomes that, once released, fuse with membranes of neighboring cells and deliver their contents from one cell to another (Schwab et al., 2015). Therefore, Nef may regulate intercellular communication through exosomes extending its immune regulation on neighboring uninfected cells (Ali et al., 2010; Konadu et al., 2015; Olivetta et al., 2016). Exosomes containing Nef have been also shown to induce cell death of resting CD4^+^ T lymphocytes, whereas no effect was observed using exosomes lacking the Nef protein (Lenassi et al., 2010). Recently, researchers have shown that HIV-1 infected CD4^+^ T lymphocytes are able to release exosomes and activate bystander quiescent human CD4^+^ T lymphocytes, thus stimulating viral spread. Of note, HIV-1 replication occured also in quiescent CD4^+^ T lymphocytes once they were treated with exosomes derived from cells expressing Nef alone, suggesting an active involvement of Nef in this mechanism (Arenaccio et al., 2014).

### A Novel Virus-Host Inter-kingdom Communication Mechanism

HIV-1 exploits intercellular vesicle trafficking both for the biogenesis of retroviral particles and infection. Thereby, the formation of new infectious particles is achieved through exosome biogenesis pathway (i.e., the Trojan exosome hypothesis) (Gould et al., 2003). Many of these exosomes contain not only proteins such as cytokines but also RNAs (mRNA, microRNAs and other small non-coding RNAs) that can be functional once entered into target cells. In particular, one of RNA species contained into exosomes are microRNAs (miRNAs). MiRNAs are an important class of small, non-coding RNAs that bind to 3′UTR of mRNA targets based on sequence complementarity and play fundamental roles during basic cellular processes such as differentiation, development and death. MiRNAs are also involved in intestinal barrier dysfunction. A recent study showed that TNFα can increase the expression of miR-122a in Caco-2 cells and in mouse small intestine. miR-122a can bind the 3′UTR of occludin mRNA leading to its degradation and to the regulation of TJ permeability (Ye et al., 2011).

Since miRNAs have been confirmed to play a role in immune cell development and have an impact on cell functions, it is undeniable that are related to autoimmune diseases (Zhang et al., 2014). In particular, an interesting issue is whether miRNAs have a role in the regulation of HIV-1 gene expression and thus have an important role in HIV-1 infection. A couple of studies emphasized the complex interaction existing among cellular and viral miRNA functions. In particular, Nef facilitates HIV-1 replication and miRNA-mediated control of Nef expression is therefore of paramount importance for its pathogenesis (Tan Gana et al., 2012; Swaminathan et al., 2014).

The HIV-1 region encompassing Nef and LTR (Nef/LTR) acts similarly to eukaryote 3′UTRs (**Figure 1**). Therefore, miRNA binding sites in this region could potentially modulate the expression of many proteins during HIV-1 replication cycle. Computational predictions revealed that the HIV-1 genes can be targeted by human miRNAs (Ahluwalia et al., 2008). Luciferase reporter assays demonstrated that hsa-miR-29a and hsa-miR-29b, expressed in human peripheral blood mononuclear cell, can bind to the region coding for the HIV-1 accessory protein Nef, decreasing the protein expression and ultimately affecting the replication of HIV-1 (Ahluwalia et al., 2008). More recently, miRNA microarray analyses on CD4^+^CD8^-^ PBMC reported that levels of hsa-miR-29a/b, hsa-miR-155 and hsa-miR-21 were significantly down-regulated, whereas hsa-miR-223 levels were upregulated upon infection. These results were validated by TaqMan miRNA qPCR and bioinformatics analysis confirmed their ability to target the HIV-1 Nef-3′LTR region (Sun et al., 2012).

**FIGURE 3:**
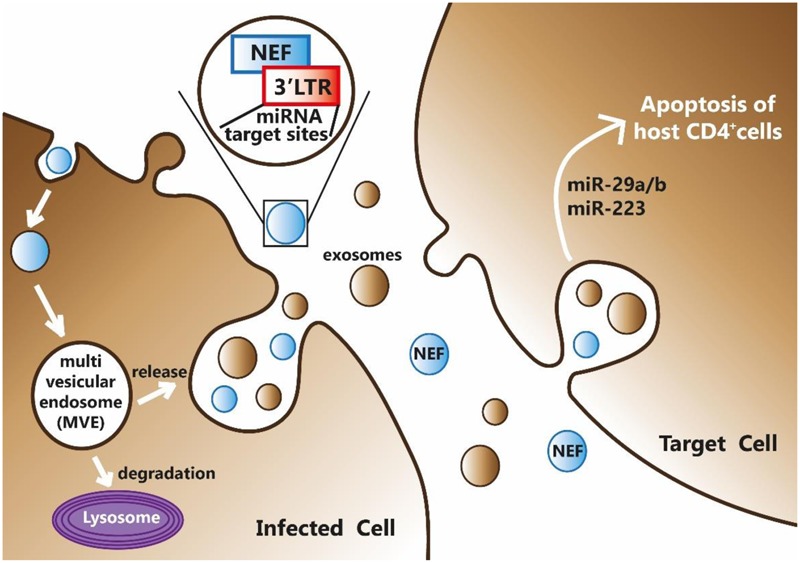
HIV-1 Nef can bind miRNAs at its 3′-end and be secreted by infected cell through exosomes formation. Exosomes, together with their miRNAs and Nef content, may enter into target cells thus mediating a potential inter-kingdom communication mechanism.

Noteworthy, HIV-1 Nef protein is not only regulated by human miRNAs, but Nef itself can modulate the expression of cellular miRNAs by promoting viral replication and progression to AIDS, and can increase the formation of MVBs. Nef interacts with Argonaute2, an integral component of the miRNA-mediated silencing pathway that localizes predominantly to endosomal membranes and in MVBs. This results in a redistribution of RNA-induced silencing complex components between cells and exosomes leading to a viral suppression of RNAi (Aqil et al., 2013). HIV-1 has been reported not only to modulate cellular miRNA profiles but also interfere with their biogenesis, likely affecting also the transport of miRNAs into exosomes. In a recent study, exosomes have been purified from human monocyte U937 cells that stably expressed Nef-enhanced yellow fluorescent Protein (EYFP) fusion protein (Aqil et al., 2014). The expression profile analysis of miRNAs contained in exosomes secreted by U937/Nef-EYFP, revealed 47 miRNAs selectively released into Nef exosomes and only two miRNAs retained in Nef-expressing cells. This suggested that Nef expression induces a secretion of many more miRNAs in exosomes compared to those that are retained in cells. Since one miRNA can potentially target hundreds of transcripts, changes in miRNA expression levels can have significant effects on many biological processes. Bioinformatics analyses can be used to identify targets and predict pathways. Using miRWalk tool the authors obtained 1,565 validated target genes for the 47 exosomal miRNAs and 107 validated mRNA target for the two miRNAs retained in Nef-expressing cells. These target genes were then subjected to pathway analysis using the DAVID Bioinformatics Resources. Several pathways involved in HIV-1 pathogenesis, came up in this analysis including TGF-beta signaling, Toll-like receptors signaling, apoptosis, cell cycle, T-cell receptor signaling, p53 signaling, mitogen activated protein kinase signaling, cytokine-cytokine receptor interaction, Janus kinase-signal transducer and activator of transcription signaling, B-cell receptor signaling, mTOR signaling and chemokine signaling (Aqil et al., 2015). Then, a genome-wide transcriptomic analysis identified four mRNAs that are exclusively retained in Nef-expressing human monocytic cells and their corresponding miRNAs that are preferentially secreted in exosomes from these cells (Quaranta et al., 2006). Overall, these results indicate that the mechanisms and biological processes where Nef is involved either for regulating miRNA expression or being regulated by, are numerous and not completely understood. Interestingly, the role of Nef is multifaceted and no simple regulatory network can be inferred to illustrate the processes involved in HIV-1 infection in humans (**Figure 3**).

As illustrated above, exosomes with their inner miRNA and Nef content might further contribute to complicate this pattern.

### Summary and Conclusion

HIV-1 Nef, expressed early during viral infection, is involved in AIDS pathogenesis and disease progression and uses multiple strategies to promote virus replication, depending on its intracellular localization (Quaranta et al., 2006). Nef interferes with cellular signal transduction pathways and modulates the cell surface expression of several receptors critical for HIV-1 infection and transmission. Moreover, it is also involved in the impairment of the immune response. HIV-1 infection is characterized by increased intestinal permeability and enteropathy. The protein triggers the local release of cytokines that lead to impairment of barrier function by altering the expression of TJ-associated proteins (Quaranta et al., 2011a,b). To date the pathobiology of HIV-1 Nef in mucosal dysfunction has still not completely elucidated.

Nef properties have been associated mainly with its biochemical activities within infected cells, from which it is secreted in association with MVBs. Following their fusion with the plasma membrane, exosomes containing Nef may be secreted. The function of exosomes in HIV-1 pathogenesis is nowadays beginning to emerge. Exosomes contain mRNAs and proteins but also miRNAs (Madison and Okeoma, 2015). We think that focusing on a high-throughput characterization of miRNAs contained in circulating extracellular vesicles and on the study of their role in modulating gene expression of target host cells is a necessary step to perform for clarifying completely the relationships with viral Nef. Nef may affect the distribution of cellular miRNAs between cells and exosomes and cause their dysregulation. To date, miRNAs profile was characterized in Nef-expressing monocytes and their secreted exosomes, revealing that the majority of miRNAs is located in the exosomes. Therefore, exosomes releasing miRNAs into microenvironment may intervene to mediate cell-to-cell communications. In turn, Nef may participate to this network modulating the expression of miRNA profile (Mathivanan et al., 2010). Up to now, no studies have been carried out to evaluate the effect of HIV-1 Nef on miRNA profiles of the intestinal mucosa. However, growing evidences from *in vitro* studies and animal models suggest that miRNAs may contribute to regulate intestinal epithelial TJ permeability, impaired also in HIV-1 infection and in inflammatory disorders such as bowel disease and celiac disease (Ye et al., 2011). Therefore, we hypothesize that the regulatory network in which Nef is implicated is multifaceted and that exosomes (containing Nef and miRNAs) are an integral part of this process. We also think that the advent of novel bioinformatics resources coupled to next-generation sequencing could help to clarify the role of Nef and microRNAs in viral infection. Finally, we think that the communication mechanism between the virus and the host, mediated by Nef and exosomes, could be a novel inter-kingdom communication ‘realm,’ where the virus conveys ‘messages’ to the host through Nef and exploits a ‘language’ that still has to be fully understood.

## Author Contributions

CF conceived the manuscript, wrote and organized most of the paragraphs, OV and MS contributed to write and revise the manuscript, AM wrote and revised many parts of the manuscript, revised the paragraph organization and contributed to finalize the manuscript.

## Conflict of Interest Statement

The authors declare that the research was conducted in the absence of any commercial or financial relationships that could be construed as a potential conflict of interest.
